# Postprandial dysfunction in fatty liver disease

**DOI:** 10.14814/phy2.15653

**Published:** 2023-04-20

**Authors:** Josephine Grandt, Anne‐Sofie H. Jensen, Mikkel P. Werge, Elias B. Rashu, Andreas Møller, Anders E. Junker, Lise Hobolth, Christian Mortensen, Christian D. Johansen, Mogens Vyberg, Reza Rafiolsadat Serizawa, Søren Møller, Lise Lotte Gluud, Nicolai J. Wewer Albrechtsen

**Affiliations:** ^1^ Gastro Unit Copenhagen University Hospital ‐ Amager and Hvidovre Hvidovre Denmark; ^2^ Novo Nordisk Foundation Center for Protein Research, Faculty of Health and Medical Sciences University of Copenhagen Copenhagen Denmark; ^3^ Department of Clinical Biochemistry Copenhagen University Hospital ‐ Bispebjerg and Frederiksberg Hospital Copenhagen Denmark; ^4^ Department of Pathology Copenhagen University Hospital ‐ Amager and Hvidovre Hvidovre Denmark; ^5^ Center for RNA Medicine, Department of Clinical Medicine Aalborg University Copenhagen Denmark; ^6^ Department of Clinical Physiology and Nuclear Medicine, Center for Functional and Diagnostic Imaging and Research Copenhagen University Hospital Hvidovre Denmark; ^7^ Institute for Clinical Medicine, Faculty of Health and Medical Sciences University of Copenhagen Copenhagen Denmark

## Abstract

Fatty liver disease has mainly been characterized under fasting conditions. However, as the liver is essential for postprandial homeostasis, identifying postprandial disturbances may be important. Here, we investigated postprandial changes in markers of metabolic dysfunction between healthy individuals, obese individuals with non‐alcoholic fatty liver disease (NAFLD) and patients with cirrhosis. We included individuals with biopsy‐proven NAFLD (*n* = 9, mean age 50 years, mean BMI 35 kg/m^2^, no/mild fibrosis), cirrhosis with hepatic steatosis (*n* = 10, age 62 years, BMI 32 kg/m^2^, CHILD A/B) and healthy controls (*n* = 10, age 23, BMI 25 kg/m^2^), randomized 1:1 to fasting or standardized mixed meal test (postprandial). None of the patients randomized to mixed meal test had type 2 diabetes (T2D). Peripheral blood was collected for 120 min. After 60 min, a transjugular liver biopsy and liver vein blood was taken. Plasma levels of glucose, insulin, C‐peptide, glucagon, and fibroblast growth factor 21 (FGF21) were measured. Postprandial peak glucose and C‐peptide were significantly increased in NAFLD, and cirrhosis compared with healthy. Patients with NAFLD and cirrhosis had hyperglucagonemia as a potential sign of glucagon resistance. FGF21 was increased in NAFLD and cirrhosis independent of sampling from the liver vein versus peripheral blood. Glucagon levels were higher in the liver vein compared with peripheral blood. Patients with NAFLD and cirrhosis without T2D showed impaired glucose tolerance, hyperinsulinemia, and hyperglucagonemia after a meal compared to healthy individual. Postprandial characterization of patients with NAFLD may be important to capture their metabolic health.

## Graphical abstract

Postprandial state is explored in patients with fatty liver disease highlighting the importance of evaluting fasting and postprandial conditions in metabolic diseases.

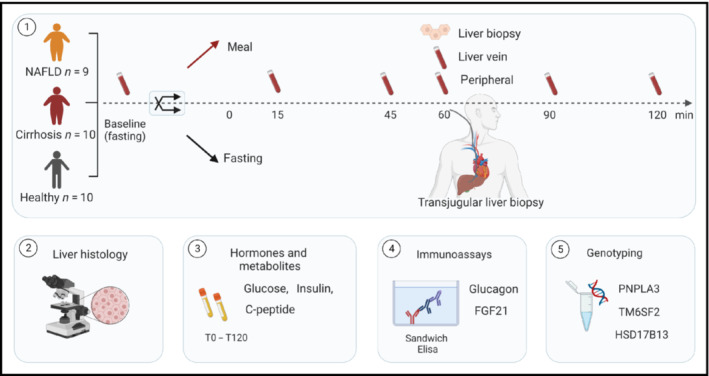

## 1 INTRODUCTION

Fatty liver disease is the most common liver disease worldwide (Younossi et al., 2018). The accumulation of triglycerides in hepatocytes can lead to metabolic and endocrine impairment of the liver. This can include a disruption of the liver alpha cell axis, a feedback mechanism between pancreatic glucagon secretion and hepatic amino acid metabolism (Wewer Albrechtsen et al., 2016). Indeed, growing evidence suggests that fatty liver disease is more a metabolic disease than isolated liver disease. Until recently, fatty liver disease was commonly distinguished by etiology as alcoholic and non‐alcoholic fatty liver disease (NAFLD). The term metabolic associated fatty liver disease (MAFLD) emphasizes the underlying systemic metabolic disturbances in most patients with fatty liver disease (Eslam et al., 2020). MAFLD may be defined as evidence of hepatic steatosis or steatohepatitis in the presence of overweight, obesity, type 2 diabetes (T2D), or at least two manifestations of metabolic dysregulation, regardless of alcohol consumption.

Most studies on patients with NAFLD are done under fasting conditions, although it is likely that impairment of the liver leads to changes in metabolism and, thereby, changes in the postprandial response. We hypothesized that metabolic dysfunction in liver disease becomes more evident when “challenged” by a meal. We therefore studied the metabolic response to a physiological, standardized liquid mixed meal compared to fasting in biopsy proven NAFLD, cirrhosis, and healthy controls in a randomized design. Our aim was to investigate changes in markers of metabolic dysfunction including plasma levels of glucose, insulin, C‐peptide, glucagon, and fibroblast growth factor (FGF21) between groups and between postprandial and fasting conditions. The nature of the study is exploratory and the primary outcome (phosphoproteomic changes at a liver tissue level) is not reported in this article.

## 2 EXPERIMENTAL PROCEDURES

### 2.1 Subjects

We recruited 30 participants: 10 healthy controls, 10 patients with NAFLD, and 10 with cirrhosis. One patient in the fasting NAFLD group was excluded as we were unable to collect a biopsy. Accordingly, 29 patients were included in the analyses. The study included a screening visit and a study day with no follow‐up visits. All visits and examinations were conducted at the Copenhagen University Hospital Hvidovre.

### 2.2 Screening

Patients in the NAFLD and cirrhosis group were consecutively recruited in the outpatient clinic at the Gastro‐Unit, Hvidovre Hospital, if found eligible for diagnostic transjugular liver biopsy by their treating physician. Healthy subjects were recruited through self‐referral by advertising. The screening visit included an interview, informed consent, transient elastography measurement (FibroScan®, Echosens), blood tests (complete blood count, liver function tests, basic metabolic tests, and cholesterol), as well as anthropometric measurements, including waist circumference and bioimpedance measurement (SECA electronics) with visceral adipose tissue (VAT) measurement. Transient elastography was performed by a trained physician and required at least ten valid measurements with an IQR <30%. All participants were fasting for at least 4 h before the measurement, and M or XL probes were used as appropriate.

### 2.3 Inclusion and exclusion criteria

Inclusion criteria for healthy controls were age 20–40 years, BMI 20–25 kg/m^2^, and no signs of fatty liver disease in transient elastography, defined as Controlled Attenuated Parameter (CAP) values <238 dB/m and liver stiffness measurement (LSM) below 7.0 kPa. Exclusion criteria were excessive alcohol intake (>14 units/ week for men and >7 units/ week for women), complete blood count, liver function tests, basic metabolic tests or cholesterol outside the normal range, chronic heart, lung, kidney, or metabolic diseases, smoking and use of medication other than oral contraceptives or mild painkiller. For the NAFLD group, we included patients from the outpatient clinic with a clinical diagnosis of fatty liver disease indicated by transient elastography during the screening visit. There were no requirements on age, BMI, medication, or T2D. Inclusion criteria for cirrhosis were a clinical diagnosis of cirrhosis, indicated by ultrasound or transient elastography or confirmed on a previous biopsy. There were no requirements regarding the etiology of cirrhosis, Child‐Pugh score, medication, or previous decompensation. Patients with ongoing alcohol abuse or malignant diseases were excluded.

### 2.4 Ethics statement

The study was conducted in compliance with the Declaration of Helsinki, and the local ethical committee approved the study (H − 18052725). The study was registered at ClinicalTrials.gov (NCT03849235). All participants gave written informed consent before inclusion.

### 2.5 Randomization

Participants were randomized 1:1 to either fasting or postprandial on study day after ensuring that participants overheld the overnight fast. The randomization sequence was created using the online website “randomizer.org,” and randomization was done by an independent study nurse not otherwise involved in the study. We used sealed, non‐see‐through envelopes, numbered from 1–10 in each group, assigned to participants in chronologically order.

### 2.6 Experimental design

**FIGURE 1:**
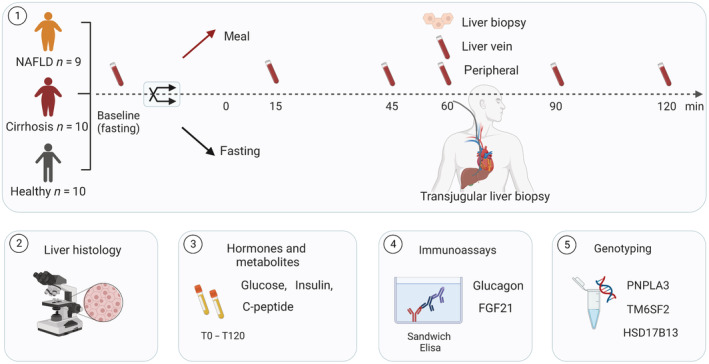
Study Design (1) Protocol Study Day: Participants were randomized to either remain fasting or consume a liquid meal. Peripheral blood samples were taken at several timepoints. Transjugular liver biopsies were performed after 60 min, and blood from the liver vein was collected. (2) Liver histology: Two expert pathologists scored biopsies and evaluated NAS scores. (3) Hormones and metabolites: We measured glucose, insulin, and C‐peptide at all timepoints. (4) Immunoassays: We measured glucagon and FGF21 at all timepoints in peripheral blood and in liver vein blood at 60 min. (5) Genotyping: We analyzed the genotypes of PNPLA3, TM6SF2 and HSD17B13. Figure created with biorender.com.

On the study day (Figure 1), a peripheral catheter was placed in the cubital vein, and baseline blood samples (Timepoint 0) were collected. After randomization, participants allocated to “postprandial” consumed a liquid meal consisting of 200 mL Nutridrink, Nutricia (300 kcal, 36.8 g carbohydrates, 11.8 g proteins, 11.6 g fat). Participants allocated to “fasting” remained fasting. Peripheral blood samples were collected after 15, 45, 60, 90, and 120 min. Transjugular liver vein catheterization was performed in all study participants to obtain liver biopsies and to measure hepatic venous pressure gradients (HVPG). At 60 min, a blood sample from the right liver vein was collected.

### 2.7 Transjugular liver biopsy and hepatic venous pressure gradient measurement

Liver vein catheterization was performed under local anesthesia by experienced specialists according to national standards. In brief, the jugular vein was cannulated under sonographic control, and a Swan‐Ganz balloon catheter was guided to the hepatic veins under fluoroscopic guidance. A blood sample was obtained from the right liver vein. The hepatic venous pressure gradient (HVPG) was measured as the wedged (WHVP) minus the free hepatic venous pressure (FHVP). Pressures were measured directly by a capacitance transducer (Simonsen & Weel). Needle biopsies were taken either by a Cook or Kimal 18 or 19 French biopsy canula. Participants were observed for 2 h after the biopsy and then discharged from the hospital.

### 2.8 Blood sample from liver vein

A blood sample from the liver vein was obtained under the transjugular liver biopsy procedure at timepoint 60 in 26 of 29 patients. No blood sample from the liver vein was obtained in one patient with NAFLD and two patients with cirrhosis.

### 2.9 Histological assessment

Two expert pathologists independently performed the histological assessment of liver biopsies in all study participants. Liver biopsies were processed using neutrally buffered formalin for 24–48 h and were embedded in paraffin. Blocks were cut at 3 μm, and slides were stained among other with hematoxylin and eosin (HE) and picro sirius red (PSR). Liver Fibrosis was staged from 0 (no fibrosis) to 4 (cirrhosis). The NAFLD activity score (NAS) was used to assess biopsies (Brunt et al., 2005). Disagreement was resolved by discussion.

### 2.10 Handling and biochemical analyses of blood samples

Peripheral and liver vein blood samples were immediately stored on ice until centrifugation at 4°C. After allocation in cryotubes, plasma samples were transported on dry ice and stored at −80 °C until analysis. Plasma concentrations of glucagon were analyzed using ELISA (Mercodia Glucagon ELISA, Mercodia AB) (Wewer Albrechtsen et al., 2014). FGF21 concentrations were quantified using Quantikine® Human FGF‐21 Immunoassay (Catalog Number DF2100). We measured insulin and C‐peptide concentrations by immunoassay with Cobas e 602.

### 2.11 Genotyping and genetic risk score

Study participants were genotyped for three variants; the patatin‐like phospholipase domain‐containing protein 3 rs738409 C > G (PNPLA3 I148M), the transmembrane 6, superfamily member 2 rs58542926 C > T (TM6SF2 E167K), and the splice variant hydroxysteroid 17‐beta dehydrogenase 13 rs72613567 T > TA (HSD17B13) variant, using TaqMan 5′‐nuclease assays in duplicate (QuantStudio 3; Thermo Fisher, Waltham, MA) (Gellert‐Kristensen, et al., 2020). Genotyping success rate was >99%.

The three genotypes were coded as 0 for noncarriers, 1 for heterozygous, and 2 for homozygous for the risk‐increasing allele. The risk‐increasing allele for PNPLA3 and TM6SF2 was the minor allele, while the risk‐increasing allele for HSD17B13 was the major allele.

### 2.12 Statistical analysis

The study was designed as an exploratory study without prespecified outcomes reported here or a statistical analysis plan. The primary aim was comparisons in markers of metabolic dysfunction between healthy individuals, obese individuals with NAFLD and patients with cirrhosis. Comparisons between the 3 study groups were calculated using analysis of variance (One‐way ANOVA) with posthoc test (Šidák) for multiple comparisons. Total area under the curve (tAUC) was calculated with the trapezoid rule. For incremental AUC (iAUC), baseline values were subtracted and iAUC was calculated including only positive values above baseline. Net AUC (nAUC) was calculated as the area of peaks above baseline, subtracted with the area of peaks below that baseline. Peak concentrations were calculated as mean ± SEM of individual peaks in the different groups. Two‐way ANOVA with meal and group as categorical fixed effects and individual subjects as random effect was used to analyze tAUC, iAUC, nAUC, and peak concentrations. A posthoc test (Šidák) for multiple comparisons was performed for differences between groups. Shapiro–Wilk test and QQ‐plots were used to test the distribution of residuals. We used Pearson correlation and simple linear regression to correlate FGF21 concentrations to clinical and biochemical patient data. GraphPad Prism Version 9.1.1 and R statistical software were used for statistical analyses and graphical illustrations. Data are expressed as mean ± SEM if not stated otherwise. *p*‐values <0.05 were considered statistically significant.

### 2.13 Definitions and calculations

MAFLD was defined as evidence of hepatic steatosis in the presence of either overweight, obesity, T2D, or at least two of the following: waist circumference > 102 cm for men and >98 cm for women, blood pressure > 135/85 mm Hg or specific drug treatment, triglycerides >1.7 mmol/L or specific drug treatment, HDL <1 mmol/L for men and <1.3 mmol/L for women or specific drug treatment, fasting glucose >5.6 mmol/L, HbA1c 39–47, HOMA‐IR > 2.5, or CRP > 2 mg/7. Overweight was defined as BMI > 25 kg/m^2^ and obesity as BMI > 30 kg/m^2^. Impaired fasting glucose (IFG) was defined as fasting glucose concentrations of 6.1–6.9 mmol/L after WHO criteria for IFG. HOMA‐IR was calculated using the formula: [ (Insulin (pmol/L)/6) * (Glucose (mmol/L))/22.5] (Matthews et al., 1985). Hepatic insulin extraction was calculated as ratio between C‐peptide (pmol/L) and insulin (pmol/L).

## 3 RESULTS

### 3.1 Patients with NAFLD and cirrhosis are characterized by obesity and impaired fasting glucose

General characteristics of study participants are presented in Table 1.

**TABLE 1:** Clinical, anthropometric and biochemical data at baseline in healthy, NAFLD and cirrhosis.

Variable	Healthy (*n* = 10)	NAFLD (*n* = 9)	Cirrhosis (*n* = 10)
Age, years	25 ± 1	50 ± 5***	62 ± 3^+++^
Male/female	5/5	4/5	5/5
BMI, kg/m^2^	23 ± 0	35 ± 2***	32 ± 2^+++^
Waist circumference, cm	80 ± 3	113 ± 4***	113 ± 4^+++^
VAT, L	0.8 ± 0.3	4.5 ± 0.6***	4.2 ± 0.7^+++^
T2D, *n*	0	0	2
Family history of T2D	2	3	2
A1c, mmol/mol	33 ± 1	34 ± 2	36 ± 4
Fasting glucose, mmol/L	5.0 ± 0.1	5.5 ± 0.2	7.2 ± 0.8^++^
C‐peptide, pM	484 ± 38	1001 ± 105*	1317 ± 204^+++^
Insulin, pM	38 ± 5	92 ± 17	163 ± 31^+++^
Glucagon, pM	6 ± 1	9 ± 2	14 ± 5
Glucagon/ Insulin ratio	0.19 ± 0.03	0.13 ± 0.03	0.13 ± 0.04
HOMA‐IR	1.4 ± 0.2	3.7 ± 0.8*	8.7 ± 1.7^+++^
Hepatic insulin extraction	13.7 ± 1.3	11.3 ± 1.0	10.1 ± 3.0
HDL, mmol/L	1.6 ± 0.1	1.3 ± 0.2	1.2 ± 0.1
Triglycerides, mmol/L	0.8 ± 0	1.5 ± 0.2*	1.4 ± 0.2^+^
ALT U/L	10 ± 1	35 ± 10*	19 ± 4
AST U/L	21 ± 1	40 ± 8	83 ± 30^+^
FGF‐21, pg/mL	90 ± 27	200 ± 35	258 ± 46^++^
FIB‐4	0.6 ± 0	1.0 ± 0.2***	6.3 ± 1.2^+++^
CAP, dB/m	186 ± 8	300 ± 18***	303 ± 12^+++^
LSM, kPa	4.5 ± 0.3	6.5 ± 0.9^###^	42.7 ± 8.0^+++^
NAS Score	0 (0–0)	2 (1–4)	3 (2–4)
HVPG, mm Hg	2 ± 0	3 ± 0^###^	11 ± 2^+++^
PNPLA3			
WT	3	2	3
Heterozygous	6	7	5
Homozygous	0	0	1
TM6SF2			
WT	7	8	5
Heterozygous	3	1	4
Homozygous	0	0	0
HSD17B13			
WT	0	0	2
Heterozygous	5	2	2
Homozygous	5	7	5

*Note*: Differences between groups are calculated using One‐Way ANOVA and corrected for multiple comparisons using Šidák‐Holm correction.

Abbreviations: A1c, Hemoglobin A1C; ALT, Alanine Transferase; AST, Aspartate Transferase; BMI; Body Mass Index; CAP, Controlled Attenuation Parameter; FGF‐21, Fibroblast‐Growth‐Factor 21; FIB‐4, Fibrosis‐4; HDL, High‐Density Lipoprotein; HOMA‐IR, Homeostatic Model Assessment for Insulin Resistance; HVPG, Hepatic Venous Pressure Gradient; LSM, Liver Stiffness Measurement; NAS Score, NAFLD Activity Score; T2D, Type 2 Diabetes; VAT, Visceral Adipose Tissue; WT, Wild Type. Data are presented as mean ± SEM.

* NAFLD vs. healthy, *p* < 0.05

*** NAFLD vs. healthy, *p* < 0.001.

^###^ NAFLD vs. cirrhosis, *p* < 0.001.

^+^ Cirrhosis vs. healthy, *p* < 0.05.

^++^ Cirrhosis vs healthy, *p* < 0.01.

^+++^ Cirrhosis vs. healthy, *p* < 0.001.

The mean age of patients was 50 ± 5 years in the NAFLD and 62 ± 3 years in the cirrhosis group. The mean BMI was 35 ± 2 kg/m^2^ in NAFLD and 32 ± 2 kg/m^2^ in cirrhosis, and seven patients with NAFLD and six patients with cirrhosis were obese. In NAFLD and cirrhosis, 8/9 and 9/10 classified as MAFLD, respectively. None of the patients in the NAFLD group had T2D. Impaired fasting glucose >6.1 mmol/L was present in two patients with NAFLD. In cirrhosis, six patients had impaired fasting glucose, and two patients allocated to fasting had T2D treated with metformin alone. In healthy individuals, the mean age and BMI were 25 ± 1 years and 23 ± 0 kg/m^2^, respectively, and healthy individuals had normal waist circumference and VAT. All healthy participants had normal liver histology with less than 5% hepatic fat content and no histological signs of fibrosis. Patients in the NAFLD group mainly had simple fatty liver disease with no or mild fibrosis except one patient with stage 3 fibrosis (median fibrosis stage; 0 (IQR 0–3), median NAS score; 2 (IQR 1–4)). The median NAS score in cirrhosis was 3 (2–4), and the etiology of cirrhosis was alcoholic liver disease (*n* = 9) or hepatitis B (*n* = 1). The patient with hepatitis B cirrhosis had virus loads below the detection limit under treatment with Entecavir and did not consume alcohol. Six patients had Child‐Pugh A, and four patients had Child‐Pugh B. The mean HVPG in cirrhosis was 11 ± 2 mmHg, with four patients having clinically significant portal hypertension defined as HVPG > 10 mm Hg. One patient in the NAFLD group was treated with statins for hypercholesterolemia, and two were treated for hypertension. In the cirrhosis group, one patient was treated with GLP‐1 receptor agonists for fatty liver disease and one with statins for hypercholesterolemia. After randomization to fasting and postprandial, there were no significant differences in gender distribution, age, BMI, glucose, lipid concentrations, liver function tests and FibroScan measurements within the 3 study groups. In cirrhosis, the distribution of patients with Child A and B cirrhosis was similar after randomization.

### 3.2 Insulin resistance and hyperglucagonemia are present in patients with NAFLD and cirrhosis

Hyperinsulinemia and hepatic insulin resistance were observed at fasting condition in patients with NAFLD and cirrhosis. C‐peptide concentrations were significantly increased in NAFLD (*p* = 0.020) and cirrhosis (*p* < 0.001) compared to healthy controls, but not statistically different between NAFLD and cirrhosis (*p* = 0.659, Table 1). The HOMA‐IR (marker of insulin resistance) was 3.7 ± 0.8 in NAFLD, and 8.7 ± 1.7 in cirrhosis, and six patients with NAFLD and six patients with cirrhosis had HOMA‐IR >2. Accordingly, we found fasting hepatic insulin extraction, calculated as ratio of C‐peptide and insulin, to be decreased in NAFLD, and lowest in cirrhosis. Patients with NAFLD and cirrhosis had fasting hyperglucagonemia, being most pronounced in cirrhosis (Table 1). The glucagon insulin ratio was decreased in NAFLD and cirrhosis compared to healthy. Triglyceride concentrations were similar in NAFLD (1.5 ± 0.2 mmol/L) and cirrhosis (1.4 ± 0.2 mmol/L).

### 3.3 Metabolic dysfunction in patients with NAFLD and cirrhosis allocated to fasting

**FIGURE 2:**
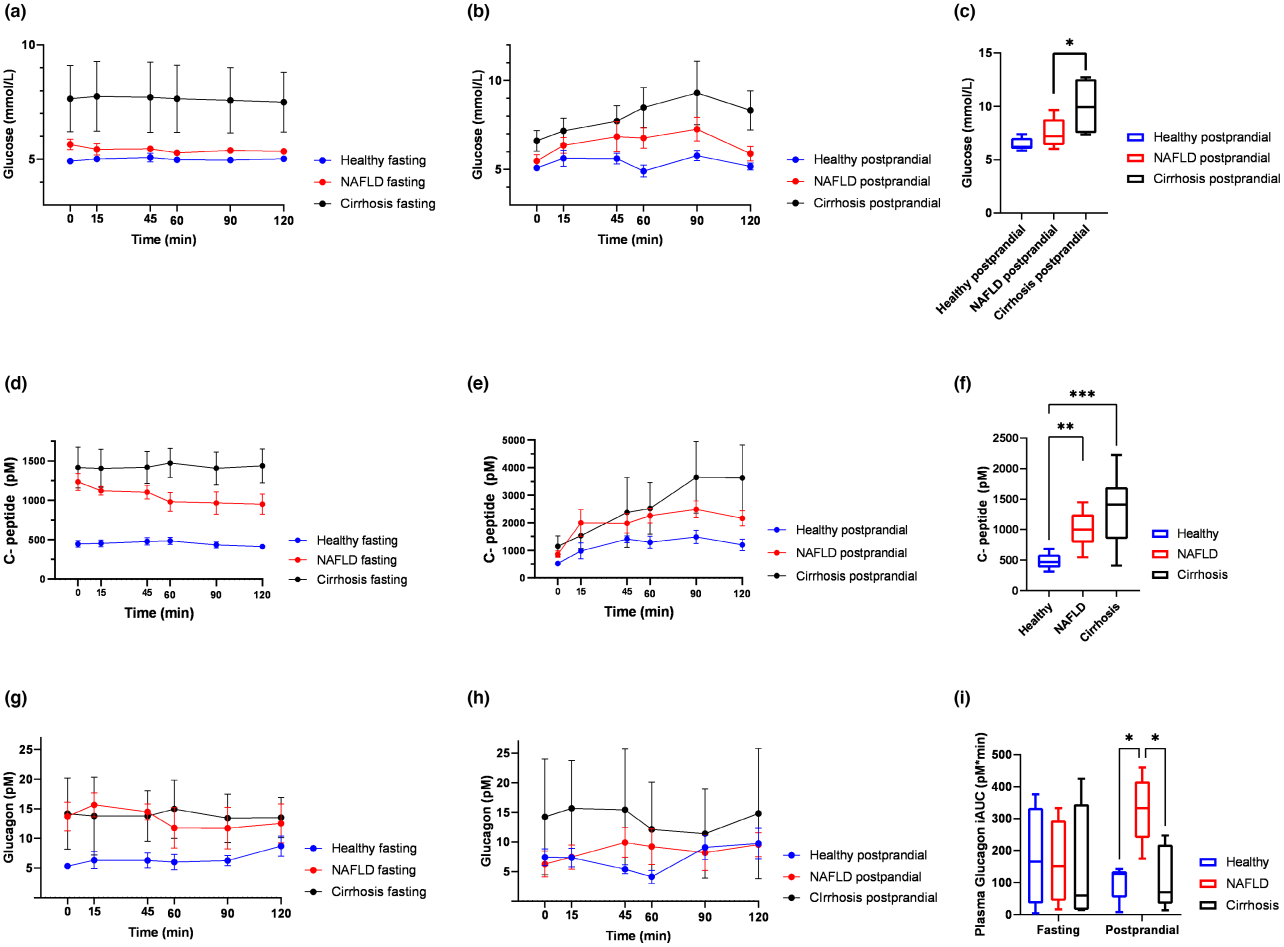
Patients with NAFLD and cirrhosis have glucagon and insulin resistance during fasting and in a postprandial state. (a) Glucose concentrations in fasting healthy, NAFLD and cirrhosis (*n* = 5–4–5) (b) Glucose concentrations in postprandial healthy, NAFLD and cirrhosis. (*n* = 5–5–5) (c): Peak glucose concentrations in postprandial healthy, NAFLD, and cirrhosis. (*n* = 5–5–5) (d): C‐peptide concentrations in fasting healthy, NAFLD and cirrhosis (*n* = 5–4–5) (e) C‐peptide concentrations in postprandial healthy, NAFLD and cirrhosis (*n* = 5–5–5) (f) Baseline C‐peptide in healthy NAFLD and cirrhosis. (*n* = 10–9–10) (g) Glucagon concentrations in fasting healthy, NAFLD and cirrhosis (*n* = 4–4–5), (h): Glucagon concentrations in postprandial healthy, NAFLD and cirrhosis (*n* = 5–5–5) (i): iAUC glucagon in fasting and postprandial healthy, NAFLD and cirrhosis (fasting, *n* = 4–4–4; postprandial, *n* = 5–5–5). Data in (a–i) are presented as mean ± SEM. Baseline and peak concentrations were compared with one‐way ANOVA. iAUCs were analyzed by two‐way ANOVA (comparing groups fasted and postprandial) and corrected for multiple testing by Holm‐Šidák correction. *p < 0.05, **p < 0.01 and ***p < 0.001.

Glucose, C‐peptide, and glucagon concentrations were as expected stable over the 120 min study period in participants allocated to fasting. Patients with cirrhosis had numerical highest glucose levels. Glucose concentrations were similar in patients with NAFLD and healthy controls, while C‐peptide concentrations were 2‐fold increased in NAFLD compared to healthy and similar to patients with cirrhosis. (Figure 2a‐f).

Glucagon concentrations were higher in patients with NAFLD and cirrhosis compared to healthy (Figure 2g‐i).

### 3.4 Metabolic dysfunction is pronounced postprandially in patients with NAFLD and cirrhosis

We found significant differences in the metabolic response to the standardized mixed meal between patients with NAFLD, cirrhosis and healthy participants, and these differences were most pronounced in patients with cirrhosis.

Postprandial glucose concentrations were higher in both NAFLD and cirrhosis compared to healthy. In NAFLD and cirrhosis, glucose increased for up to 90 min after the meal intervention while concentrations normalized to baseline within 60 min in healthy. Postprandial iAUC for glucose was higher in NAFLD and cirrhosis compared to healthy, with a 2.8‐fold increase in NAFLD and a 3.8‐fold increase in cirrhosis. These findings indicate impaired postprandial glucose regulation in NAFLD and cirrhosis, although none of the patients in the postprandial groups had T2D. However, likely reflecting that these individuals may be characterized as having pre‐diabetes. When investigating peak glucose concentrations in the groups, we found highest peak concentrations in cirrhosis that were statistically significantly different from NAFLD and healthy. (Table 2; Figure 2c). We measured postprandial C‐peptide concentrations to evaluate pancreatic insulin production. C‐peptide concentrations increased more in NAFLD and cirrhosis postprandially. Thus, NAFLD and cirrhosis patients reached peak C‐peptide concentrations of 2675 ± 273 pM and 3340 ± 1048 pM, respectively, compared with 1689 ± 190 pM in healthy. The total and incremental AUC for C‐peptide showed both a 1.7‐fold increase in NAFLD and cirrhosis compared with healthy. NAFLD and cirrhosis had a similar postprandial increase in C‐peptide. Although baseline insulin concentrations were highest in cirrhosis, patients with NAFLD showed the greatest postprandial increase in insulin (Table 2). Total and incremental AUC for postprandial insulin concentrations were highest in NAFLD. The difference in iAUC was statistically significant compared with healthy (*p* = 0.037, Table 2). These findings indicate that postprandial hepatic insulin extraction was lowest in NAFLD. In healthy, glucagon concentrations initially decreased postprandially and re‐increased after 60 min. The same trend in glucagon concentration was seen in cirrhotic patients, which tended to decrease postprandially, although slower than in healthy. Interestingly, glucagon concentrations seemed to increase in NAFLD rather than decrease (Figure 2h). The iAUC for postprandial glucagon concentrations was significantly higher in NAFLD compared to healthy (*p* = 0.026) and cirrhosis (*p* = 0.037).

**TABLE 2:** Total AUC, incremental AUC, and peak concentrations in fasting and postprandial study groups for glucose, C‐peptide, insulin, glucagon and FGF21.

	Fasting			Postprandial			ANOVA	
	Healthy	NAFLD	Cirrhosis	Healthy	NAFLD	Cirrhosis	Meal	Group
Glucose tAUC mmol/L* min	600 ± 13	566 ± 84	917 ± 176	652 ± 19	796 ± 61	819 ± 208	0.548	0.131
Glucose iAUC mmol/L* min	12 ± 4	81 ± 78	10 ± 5	51 ± 9	141 ± 42	192 ± 64	**0.012**	0.137
Peak glucose mmol/L	5 ± 0	5 ± 0	8 ± 2	6 ± 0	7 ± 1	10 ± 1^+^	0.053	**0.006**
Insulin tAUC pM* min	4932 ± 769	13,740 ± 1983	21,071 ± 2970	16,458 ± 4942	35,209 ± 5862	28,834 ± 21,263	**0.037**	0.092
Insulin iAUC pM* min	470 ± 337	53 ± 53	2244 ± 1257	12,909 ± 3560	30,440 ± 5458*	19,286 ± 12,582	**<0.001**	0.210
Peak insulin pM	52 ± 10	128 ± 0	200 ± 43	262 ± 46	475 ± 86	493 ± 236	**0.007**	0.131
C‐peptide iAUC pM* min	2050 ± 1114	7518 ± 7518	9854 ± 5609	88,394 ± 12,499	149,873 ± 16,324	149,518 ± 70,338	**<0.001**	0.306
C‐peptide tAUC pM* min	54,501 ± 4488	108,587 ± 24,708	171,121 ± 25,112	150,794 ± 19,542	253,145 ± 29,319	253,198 ± 116,006	**0.008**	0.058
Peak c‐peptide pM	505 ± 48	669 ± 0	1410 ± 315	1689 ± 190	2675 ± 273	3340 ± 1048	**0.002**	0.052
Glucagon iAUC pM* min	178 ± 78	164 ± 65	140 ± 97	101 ± 24	329 ± 46*	11,544^#^	0.666	0.116
Glucagon tAUC pM* min	778 ± 91	1370 ± 364	1669 ± 565	885 ± 150	1035 ± 228	1384 ± 909	0.669	0.395
tAUC Glucagon Insulin ratio	22 ± 3	16 ± 2	8 ± 4	10 ± 3	5 ± 2	4 ± 3	**0.002**	**0.011**
nAUC Glucagon Insulin ratio	4 ± 3	2 ± 2	‐1 ± 3	−11 ± 5	−7 ± 4	−7 ± 6	**0.010**	0.554
FGF‐21 tAUC pM* min	11,496 ± 3004	21,355 ± 6421	39,126 ± 6390	6285 ± 3973	21,240 ± 5936	15,571 ± 834	0.056	**0.015**
FGF‐21 iAUC pM* min	1142 ± 892	3215 ± 3048	24,201 ± 943	0 ± 0	804 ± 511	786 ± 447	0.162	0.556

	Healthy fasting + Healthy postprandial	NAFLD fasting + postprandial	Cirrhosis fasting + postprandial	One‐way ANOVA, *p* value
FGF‐21 tAUC pM* min	9180 ± 2444	21,291 ± 4082	32,396 ± 6193^++^	**0.004**

*Note*: Data are presented as mean ± SEM. Two‐way ANOVA was used to test differences between healthy NAFLD and cirrhosis for fasting and postprandial. Šidák‐Holm algorithm was used for correction for multiple comparisons. One‐way ANOVA with Šidák‐Holm correction was used for comparing FGF21 concentrations in healthy, NAFLD, and cirrhosis. Abbreviation: FGF21, Fibroblast‐growth‐factor 21.

* Healthy vs. NAFLD, *p* < 0.05

^#^ NAFLD vs. cirrhosis, *p* < 0.05.

^+^ Healthy vs. cirrhosis, *p* < 0.05.

^++^ Healthy vs. cirrhosis, *p* < 0.01.

### 3.5 FGF21 is increased in patients with NAFLD and cirrhosis compared to healthy independent of postprandial status

**FIGURE 3:**
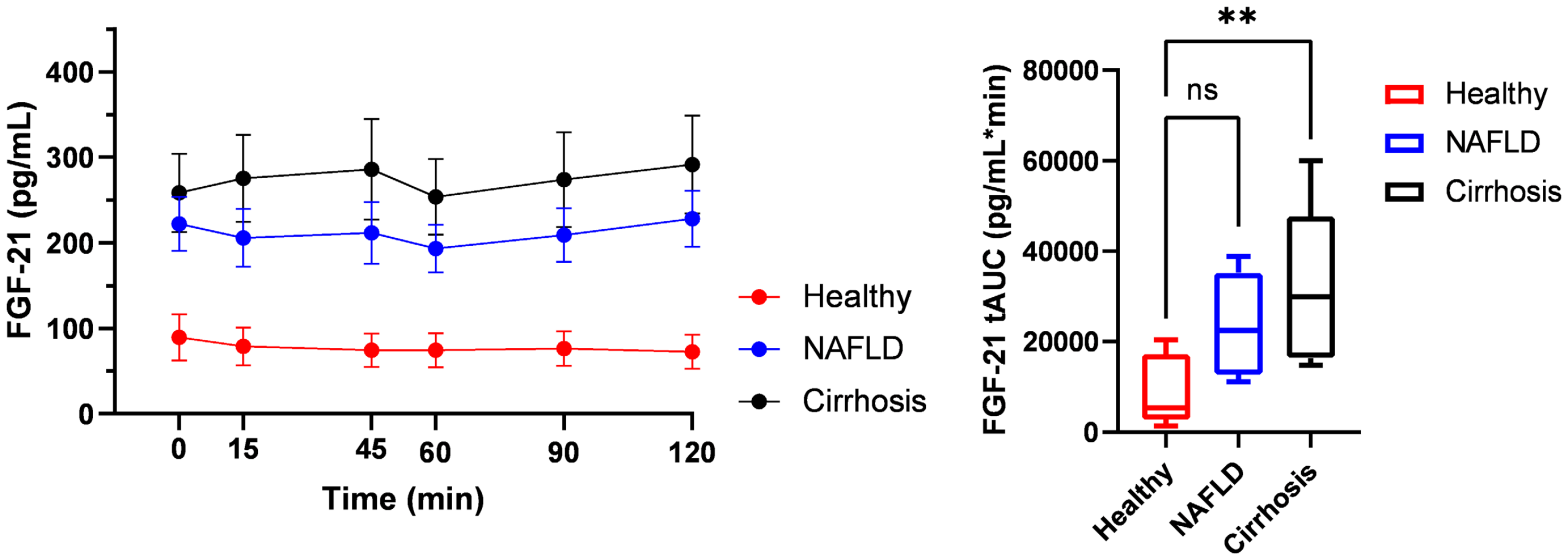
FGF21 concentrations are increased in NAFLD and cirrhosis compared to healthy. FGF21, Fibroblast‐Growth‐Factor 21. Data is presented as mean ± SEM. Total AUC for healthy, NAFLD and cirrhosis was compared using one‐way ANOVA with Šidák‐Holm correction. *p < 0.5, **p < 0.01.

Healthy participants had mean baseline FGF21 concentrations of 90 ± 7 pg/mL. Baseline FGF21 concentrations were higher in NAFLD (200 ± 35 pg/mL, 2.2‐fold increase) and highest in cirrhosis (258 ± 46 pg/mL, 2.9‐fold increase, *p* = 0.005, Table 2). We found no change in postprandial FGF21 concentrations after the meal intervention (Figure 3). Likewise, tAUC and iAUC were similar between fasting and postprandial study groups. Since the meal intervention had no significant effect on FGF21 concentrations, fasting and postprandial intervention groups were merged and presented together (Figure 3). We calculated tAUC to compare concentrations of FGF21 between groups. NAFLD and cirrhosis had higher tAUC compared with healthy, but the difference was only statistically significant between healthy and cirrhosis (*p* = 0.003) (Figure 3). We then tested the association of FGF21 levels to clinical and metabolic outcomes.

FGF21 was moderately positively associated with age (*r* = 0.61, *p* = 0.001), fasting glucose (*r* = 0.54, *p* = 0.006), waist circumference (*r* = 0.51, *p* = 0.012 *p* = 0.01) and BMI (*r* = 0.42, *p* = 0.042), but not with glucagon (*r* = 0.21, *p* = 0.347).

### 3.6 Glucagon concentrations are higher in liver vein blood than in peripheral blood

**FIGURE 4:**
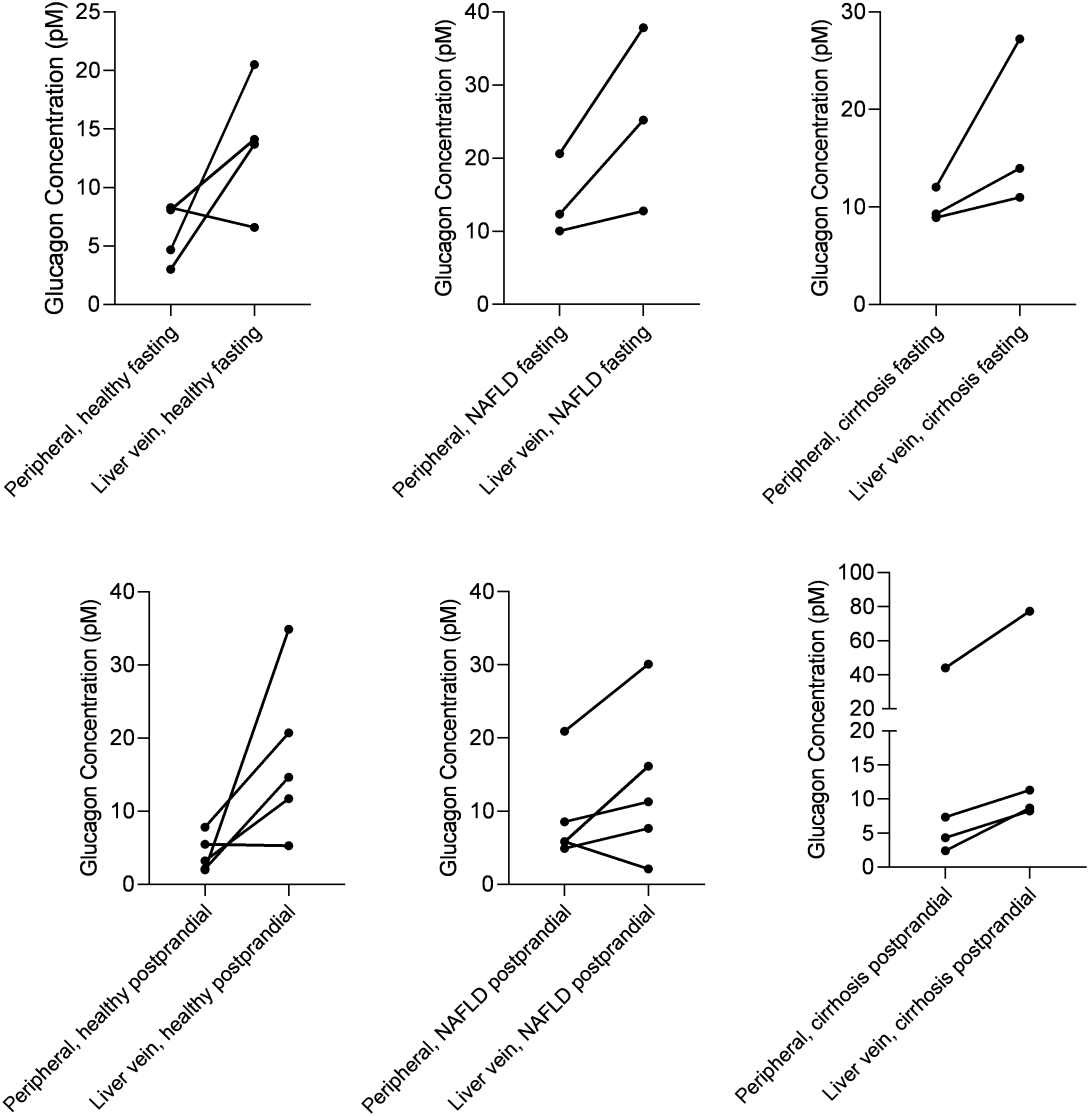
Glucagon concentrations were higher in liver vein than in peripheral blood samples Glucagon levels in peripheral versus liver vein blood in healthy, NAFLD and cirrhosis.

Glucagon concentrations measured in the liver vein blood were higher than in peripheral blood in all study groups (Table 3), although only statistically significant for healthy postprandial (*p* = 0.040). Spaghetti plots in Figure 4 indicate high inter‐subject variability. There was a significant difference between subjects (*p* < 0.001) and for sample site (peripheral vs. liver vein, *p* < 0.001).

**TABLE 3:** Glucagon concentrations were higher in liver vein blood compared to peripheral vein blood.

	Healthy fasting		Healthy postprandial		NAFLD fasting		NAFLD postprandial		Cirrhosis fasting		Cirrhosis postprandial		Two‐way‐ ANOVA		
Sample	Peripheral	Liver	Peripheral	Liver	Peripheral	Liver	Peripheral	Liver	Peripheral	Liver	Peripheral	Liver	Peripheral vs. liver	Group	Subject
FGF‐21 (pM)	95 ± 25	95 ± 22	48 ± 31	55 ± 35	194 ± 66	171 ± 40	194 ± 38	204 ± 43	272 ± 38	296 ± 47	127 ± 10	142 ± 3	0.38	**0.004**	**<0.001**
Glucagon (pM)	6 ± 1	14 ± 3	4 ± 1	17 ± 5	14 ± 3	25 ± 7	9 ± 3	13 ± 5	10 ± 1	17 ± 5	14 ± 10	26 ± 17	**<0.001**	0.741	<0.001

*Note*: Glucagon and FGF21 in liver vein and peripheral blood samples. Data are presented as mean ± SEM. Two‐way ANOVA was used to test differences between healthy, NAFLD, and cirrhosis for fasting and postprandial, subjects and peripheral blood vs. liver vein blood. Šidák‐Holm algorithm was used to correct for multiple comparisons. Abbreviation: FGF21, Fibroblast Growth Factor 21.

### 3.7 FGF21 concentrations are similar in liver and peripheral vein blood

**FIGURE 5:**
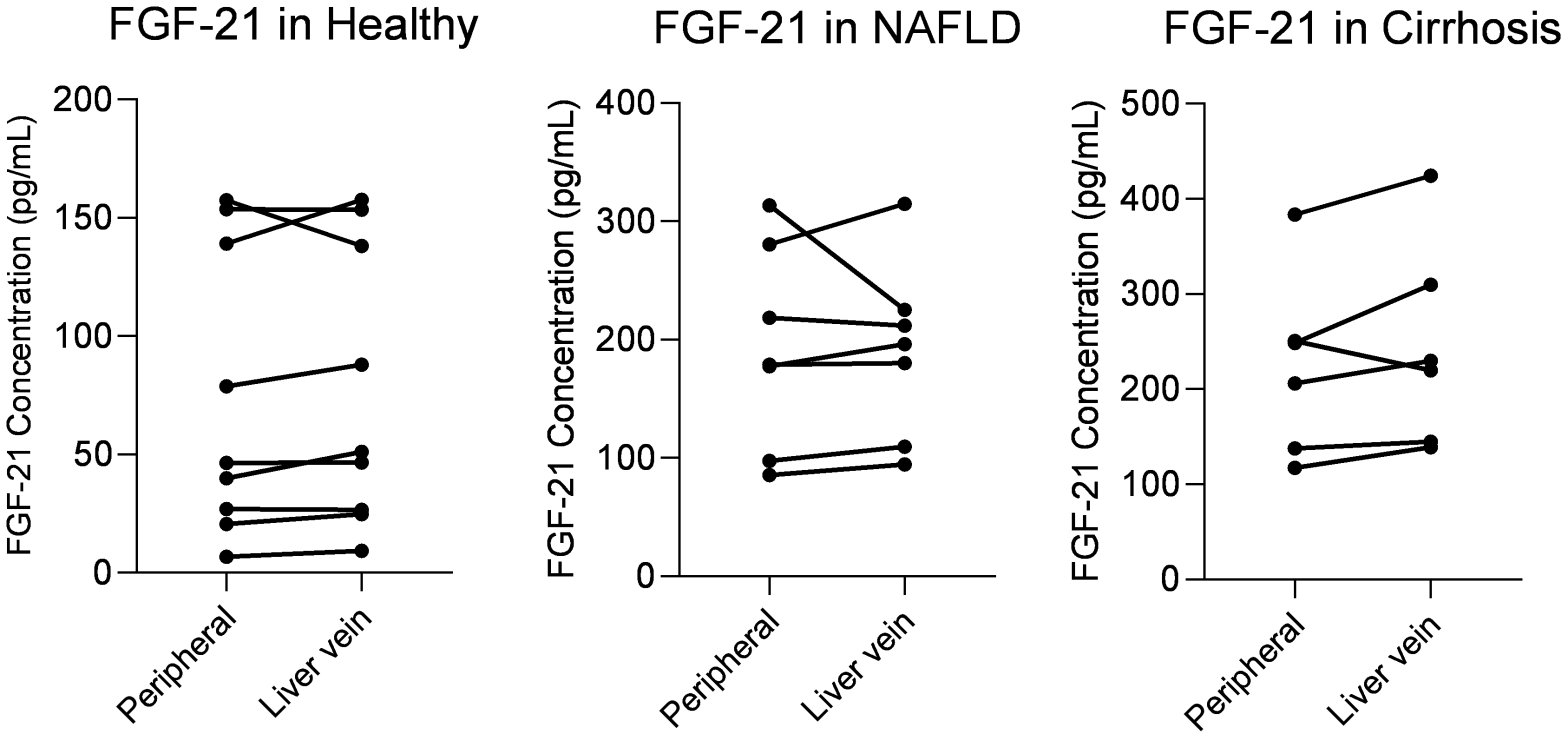
FGF21 concentrations were similar in peripheral and liver vein blood samples. FGF21 concentrations in peripheral versus liver vein blood in healthy, NAFLD, and cirrhosis.

FGF21 concentrations were similar in liver vein compared to peripheral vein blood samples (Figure 5). There was statistically significant differences between healthy, NAFLD and cirrhosis groups both when comparing FGF21 levels measured in peripheral and liver vein samples (*p* = 0.002).

## 4 DISCUSSION

In this study, we investigated fasting and postprandial metabolic and hormonal disturbances in a cohort of well‐characterized patients with NAFLD and cirrhosis, as well as normal weight, metabolic healthy young adults, both women, and men. Our patients with NAFLD and cirrhosis were overweight or obese and could be classified as having MAFLD.

The main findings were that patients with NAFLD and patients with cirrhosis exhibit the following: (1) hyperinsulinemia, hyperglucagonemia, and impaired glucose tolerance due to decreased insulin sensitivity, (2) exaggeration of these metabolic disturbances postprandially, and 3. increased levels of FGF‐21, indicating potential metabolic stress.

Firstly, our NAFLD and cirrhosis patients presented with significant metabolic dysfunction at baseline. We found pronounced metabolic disturbances in these groups, although the NAFLD cohort consisted of patients with fatty liver disease without T2D, and in cirrhosis. History of T2D was present in two patients allocated to fasting, therefore unlikely influencing postprandial results. However, as the groups were not matched on age and BMI, the present findings may reflect impaired glucose homeostasis (present in the cirrhosis group) and or overweight in the NAFLD group. Metabolic dysfunction in our cohort was most augmented in patients with cirrhosis. Metabolic dysfunction presented as hyperinsulinemia, insulin resistance assessed by HOMA‐IR, decreased hepatic insulin extraction, and hyperglucagonemia as sign of glucagon resistance.

Hyperinsulinemia and insulin resistance were previously found in patients with fatty liver disease without T2D and non‐alcoholic steatohepatitis (NASH) without obesity (Sanyal et al., 2001). Here, we demonstrated the presence of hyperinsulinemia and insulin resistance in patients with mostly simple steatosis and obesity without T2D. The NAS score as a surrogate for disease severity and presence of NASH was low in our NAFLD patients, with the highest being NAS 4. Most patients had no fibrosis, except one with fibrosis stage 2 and one with fibrosis stage 3.

In cirrhosis, impaired liver function has been found to contribute to the development of hyperinsulinemia and insulin resistance (Armandi et al., 2021). Moreover, studies have found that cytokines from the liver, excreted in reaction to the chronic inflammatory state, can induce insulin resistance in patients with cirrhosis (Armandi et al., 2021; Cai et al., 2005). Portal hypertension is another suspected culprit in developing hyperinsulinemia in cirrhosis, as portosystemic shunts were found to decrease hepatic insulin extraction (Bosch et al., 1984). In our cirrhosis group, the portal pressure was increased, and four patients had significant clinical portal hypertension. We further suggest that high BMI and the presence of MAFLD contributed to the development of metabolic disturbances in the cirrhosis group.

We found increased concentrations of glucagon in both NAFLD and cirrhosis patients. Glucagon dysregulation and hyperglucagonemia were previously associated with pre‐diabetes and T2D (Demant et al., 2018; Ichikawa et al., 2019; Rohrer et al., 2012). A growing body of evidence suggests that hepatic steatosis might be the driver of the development of hyperglucagonemia (Lake et al., 2015; Richter et al., 2022; Winther‐Sørensen et al., 2020). Here, steatosis might—through hepatic glucagon resistance along with impaired hepatocyte function—lead to the disruption of the liver alpha cell axis, a direct feedback mechanism between hepatic amino acid metabolism and pancreatic glucagon secretion. The finding of hyperglucagonemia in patients with cirrhosis has been described in several studies (Junker et al., 2015; Raddatz et al., 2004; Yoshida et al., 1998) and proposed to be related to impaired glucagon receptor signaling in the liver (Dean et al., 2017; Longuet et al., 2013).

Secondly, we found an exaggerated postprandial metabolic response after the meal intervention in both patients with NAFLD and cirrhosis. The test meal revealed increased postprandial glucose, insulin, and C‐peptide concentrations, and decreased hepatic insulin extraction and glucagon insulin ratio.

One study demonstrated insulin resistance after an oral glucose tolerance test (OGTT) in non‐diabetic patients with NASH (Pagano et al., 2002), which further underlines the presence of metabolic impairment in liver disease that might first be uncovered postprandially. Hyperinsulinemia and insulin resistance have also been found after an OGTT in patients with cirrhosis, and interestingly, that was independent of the etiology of cirrhosis (Müller et al., 1992). Moreover, we found decreased glucagon‐insulin ratios in both patient groups. A lower insulin‐glucagon ratio was previously found to be associated with the severity of NAFLD and T2D (Moh Moh et al., 2019).

Thirdly, patients with NAFLD and cirrhosis had high levels of FGF21. Compared with healthy, FGF21 concentrations were more than twice as high in patients with NAFLD and even higher in cirrhosis, although the difference between NAFLD and cirrhosis was not statistically significant. We found a positive correlation between FGF21 concentrations to age, fasting glucose, waist circumference, and BMI. Previous studies reported FGF21 to be associated with age (Hanks et al., 2015; Herpich et al., 2021), obesity (Zhang et al., 2008), and in NAFLD with the degree of steatosis (Yilmaz et al., 2010). Interestingly, while FGF21 is known to be increased in cirrhosis and alcoholic liver disease (Wagner‐Skacel et al., 2021), it was found not to be associated with disease severity assessed by Child‐Pugh score, kidney‐ or liver function, or portal pressure (Krautbauer et al., 2018). The evidence of FGF21 concentrations being mostly driven by age and metabolic factors might also explain our finding of similar FGF21 concentrations in NAFLD and cirrhosis, despite tremendous differences in disease severity. We found no response of FGF21 concentrations on the meal intervention. Indeed, FGF21 was previously found to be stimulated by glucose but not in response to a mixed meal (Vienberg et al., 2017). Furthermore, we found significantly higher glucagon concentrations in the liver vein than in the peripheral vein, with no difference between the groups. On the contrary, there was no difference in FGF21 concentrations between liver and peripheral vein blood samples.

Genetics account for approximately half of the interindividual variation in the risk of fatty liver disease. We investigated the genotypes of PNPLA3, TM6SF2, and HSD17B13 in our study cohort. For each of the three variants, individuals that are homozygous for the risk increasing alleles have at least twice the risk of developing fibrosis, cirrhosis and HCC compared to those without risk alleles. One patient with cirrhosis was homozygous for the risk increasing allele of PNPLA3 (I148M) while the distribution of the other genotypes for PNPLA3 was very similar between groups. PNPLA3 is strongly associated with hepatic fat content and liver enzymes and is the strongest common genetic risk factor for fatty liver disease (Romeo et al., 2008; Stender & Romeo, 2020). In a large population based study in 5662 individuals with median follow‐up of 23 years, the homozygous genotype for the risk‐increasing allele of PNPLA3, that was present in 12% of the cohort, was associated with an 18‐fold increased risk of liver‐related death (Stender & Loomba, 2020; Unalp‐Arida & Ruhl, 2019). The distribution of TM6SF2 alleles was similar in our cohorts, and none of the participants was homozygous for the risk increasing allele. The risk increasing allele of TM6SF2 increases the hepatic triglyceride content, promotes fibrosis and HCC and reduces the risk for cardiovascular disease (Kozlitina et al., 2014; Luo et al., 2022). The most common variant in all three study groups was homozygous for the protecting allele of HSD17B13. Homozygous carriers of the HSD17B13 variant have only half the risk of developing NASH, cirrhosis and HCC compared with wild type. The total genetic risk score calculated from all three variants was similarly low in all study groups. One could expect that genetic risk scores would be higher in those that have developed NAFLD and cirrhosis, but our small sample size is probably not sufficient to detect any trends. Our study has several strengths and limitations. One major strength of the study is that NAFLD was diagnosed by liver biopsy, the gold standard for diagnosing fatty liver disease, and two independent expert pathologists assessed the biopsies. Another strength of the study is the inclusion of healthy participants with normal liver histology as the control group. Thereby, we were able to elucidate the whole spectrum from health to severe liver disease in both fasting and postprandial.

Our study has limitations typically seen in pilot studies. Firstly, the study population was small. This reduced the statistical power of our results, and several differences observed between the study groups did not reach statistical significance. The small study population was mainly due to the pilot character of this new study design, and more participants should be included in future trials. Secondly, our healthy control group was not matched for BMI and age. NAFLD and cirrhosis patients were significantly older than healthy, and obesity was present in 60%. It is difficult to assess how much our results are driven by obesity or age alone. Therefore, it might be of interest to include an additional control group of older, obese without fatty liver disease in future studies.

In our study, we could demonstrate that a meal intervention can reveal otherwise hidden metabolic changes. These findings suggest the need for future studies to focus more on the postprandial state instead of fasting conditions. Many studies use an OGTT to measure the metabolic response in different patient groups, but this may not be the most suitable intervention to assess physiological postprandial changes. Instead, we used a standardized meal containing carbohydrates, proteins, and fat in physiological amounts to assess metabolic changes under real‐life conditions.

Most patients with cirrhosis were obese and classified as MAFLD, although the etiology of cirrhosis in our cohort consisted mainly of alcoholic liver disease, apart from one patient with hepatitis B. Hence, our study population was relatively homogenous and presented with similar degrees of glucogenic dysregulation after the meal. With MAFLD cirrhosis currently on the rise, we expect a change in the “typical cirrhotic patient,” with more patients with cirrhosis and obesity in the future. Therefore, it is of great importance to study this specific patient group.

Lastly, patients diagnosed with simple steatosis without fibrosis and NASH are often not followed‐up by a specialist. Here, we showed that patients with mostly mild MAFLD showed surprisingly pronounced metabolic disturbances and insulin resistance after a test meal, suggesting that more research needs to be conducted in this patient group. Insulin resistance is a major player in the development and progression of NASH, fibrosis, and, of course, T2D. Therefore, we suggest that patients with MAFLD should be assessed metabolically in a postprandial state rather than fasted to detect impaired glucose tolerance and pre‐diabetes and might profit from regular follow‐up to prevent progression to T2D, NASH, and fibrosis.

## 5 CONCLUSIONS

In this study, we found insulin resistance and hyperglucagonemia in patients with MAFLD without diabetes, as well as in patients with MAFLD and cirrhosis. Patients with MAFLD showed significant glucoregulatory disturbances and insulin resistance in response to a standardized meal, indicating distinct postprandial metabolic dysfunction and a condition of pre‐diabetes in these patients. Moreover, we found increased FGF21 concentrations in MAFLD that were positively associated with age, fasting glucose, and BMI. Additional studies are needed to investigate postprandial metabolic dysfunction in patients with MAFLD.

### AUTHOR CONTRIBUTIONS

Conception and Design: Nicolai J. Wewer Albrechtsen, Lise Lotte Gluud. Provision of study materials or patients: Nicolai J. Wewer Albrechtsen, Søren Møller, Lise Lotte Gluud, Anders E. Junker, Lise Hobolth, Christian Mortensen, Mikkel P. Werge, Elias B. Rashu, Andreas Møller. Collection and assembly of data: Josephine Grandt, Anders E. Junker, Mikkel P. Werge, Elias B. Rashu. Analysis and interpretation of data: Nicolai J. Wewer Albrechtsen, Lise Lotte Gluud, Josephine Grandt, Anders E. Junker, Anne‐Sofie H. Jensen, Christian D. Johansen. Drafting the article or revising it critically: All authors. Final approval of the manuscript: All authors.

### FUNDING INFORMATION

The study and Josephine Grandt, Anne‐Sophie H. Jensen, and Nicolai J. Wewer Albrechtsen were supported by the Novo Nordisk Foundation Excellence Emerging Investigator Grant—Endocrinology and Metabolism (Application No. NNF19OC0055001), the European Foundation for the Study of Diabetes Future Leader Award (NNF21SA0072746) and Independent Research Fund Denmark, Sapere Aude (1052‐00003B). The Novo Nordisk Foundation Center for Protein Research is supported financially by the Novo Nordisk Foundation (NNF14CC0001).

## Supporting information


Appendix S1.
