# Supplementary material for: Postprandial dysfunction in fatty liver disease
Source: Physiol Rep. 2023 Apr 20;11(8):e15653. doi: 10.14814/phy2.15653 (PMC10116394; doi:10.14814/phy2.15653)
Supplement: Supplementary file 1 — Appendix S1. [file PHY2-11-e15653-s001.docx]

**APPENDIX**

**Supplementary table 1:** General characteristics of participants after randomization

|  | HEALTHY  n=10 | | NAFLD  n=9 | | CIRRHOSIS  n=10 | | |
| --- | --- | --- | --- | --- | --- | --- | --- |
|  | **FASTING** | **POSTPRANDIAL** | **FASTING** | **POSTPRANDIAL** | **FASTING** | **POSTPRANDIAL** | |
| Number (men/women) | 5  (2/3) | 5  (3/2) | 4  (2/2) | 5  (2/3) | 5  (2 / 3) | 5  (3/ 2) | |
| Age, years | 23  ±1 | 26  ± 1 | 49  ±5 | 49  ±9 | 66  ± 3 | 57  ±6 | |
| BMI, kg/m^2^ | 22  ±1 | 24  ±1 | 37  ±2 | 34  ±3 | 32  ±2 | 33  ±3 | |
| Waist circumference,  cm | 75  ±3 | 84  ±4 | 118  ±5 | 109  ±6 | 116  ±3 | 111  ±7 | |
| VAT, L | 0.6  ±0.5 | 1.0  ±0.6 | 5.1  ±1.0 | 3.9  ±0.8 | 4.1  ±0.5 | 4.4  ±1.4 | |
| Obese  BMI >30, n | 0 | 0 | 4 | 3 | 3 | 3 | |
| T2DM | 0 | 0 | 0 | 0 | 2 | 0 | |
| Fam. history T2DM, n | 1 | 1 | 1 | 2 | 1 | 0 | |
| A1c | 33  ±1 | 34  ±1 | 32  ±5 | 36  ±2 | 40  ±5 | 31  ± 4 | |
| Glucose, mmol/L | 4.9  ±0.1 | 5.1  ±0.2 | 5.6  ±0.2 | 5.5  ± 0.3 | 7.6  ±1.4 | 6.6  ±0.6 | |
| Insulin,  pM | 39  ±5 | 37  ±12 | 126  ±15 | 57  ±4 | 166  ±29 | 158  ±72 | |
| C-peptide, pM | 447  ±38 | 520  ±66 | 1234  ±107 | 861  ±120 | 1416  ±260 | 1152  ±376 | |
| HOMA-IR | 1.4  ±.2 | 1.4  ±.5 | 5.3  ±.8 | 2.1  ±.1 | 9.6  ±1.9 | 7.4  ±3.4 | |
| Glucagon, pmol/L | 5  ±0 | 7  ±1 | 14  ±3 | 6  ±2 | 14  ±6 | 14  ±10 | |
| Glucagon / Insulin  ratio | 0.15  ±0.02 | 0.22  ±0.06 | 0.11  ±0.03 | 0.15  ±0.06 | 0.07  ±0.05 | 0.15  ±0.06 | |
| HDL, mmol/L | 1.6 ±0.1 | 1.6  ±0.1 | 1.4  ±0.4 | 1.3  ±0.1 | 1.1  ±0.1 | 1.2 ±0.3 | |
| Triglycerides,  mmol/L | 0.7  ±0.1 | 0.8  ±0 | 1.8  ±0.5 | 1.3  ±0.1 | 1.3  ±0.2 | 1.6  ±0.4 | |
| ALT U/L | 15  ±2 | 20  ±4 | 54  ±18 | 33  ±10 | 45  ±12 | 32  ±8 | |
| AST U/L | 24  ±2 | 27  ±2 | 40  ±6 | 22  ±1 | 78  ±27 | 113  ±49 | |
| FGF-21  (pg/mL) | 106  ±38 | 68  ±42 | 217  ±60 | 189  ±49 | 308  ±47 | 134  ±7 | |
| ALT  (U/L) | 8  ±1 | 12  ±3 | 45  ±20 | 20  ±10 | 21  ±5 | 15  ±7 | |
| AST  (U/L) | 20  ±1 | 23  ±3 | 48  ±12 | 31  ±9 | 72  ±23 | 99  ±71 | |
| FIB-4 | 0.6  ±0.1 | 0.6  ±0.1 | 1.2  ±0.4 | 0.8  ±0.3 | 7.0  ±2.3 | 5.6  ±1.2 | |
| CAP,dB/m | 182  ±17 | 190  ±6 | 316  ±35 | 289  ±18 | 304  ±17 | 302  ±20 | |
| LMS, kPa | 4  ±0 | 5  ±0 | 7  ±2 | 6  ±1 | 41  ±11 | 45  ±12 | |
| Child Pugh  A B C |  |  |  |  | 3  2  - | 3  2  - | |
| HVPG, mmHG | 2  ±1 | 2  ±0 | 3  ±1 | 2  ±0 | 8  ±1 | 14  ±3 |  |

Data are presented as mean ±SEM.
